# Influence of Leadership on Human–Artificial Intelligence Collaboration

**DOI:** 10.3390/bs15070873

**Published:** 2025-06-27

**Authors:** Rodrigo Zárate-Torres, C. Fabiola Rey-Sarmiento, Julio César Acosta-Prado, Nelson Alfonso Gómez-Cruz, Dorys Yaneth Rodríguez Castro, José Camargo

**Affiliations:** 1 Colegio de Estudios Superiores de Administración—CESA, Bogotá 110311, Colombia; rodrigo.zarate@cesa.edu.co (R.Z.-T.); fabiola.rey@cesa.edu.co (C.F.R.-S.); nelson.gomez@cesa.edu.co (N.A.G.-C.); dorys.rodriguez@cesa.edu.co (D.Y.R.C.); jose.camargo@cesa.edu.co (J.C.); 2 Departamento de Ingeniería, Pontificia Universidad Católica del Perú, Lima 15088, Peru

**Keywords:** leadership, artificial intelligence, human intelligence, hybrid interaction, technological governance

## Abstract

This study proposes a conceptual model that explains the influence of leadership on the relationship between human intelligence (HI) and artificial intelligence (AI). A qualitative, non-systematic literature review was conducted in Scopus and Web of Science of the literature published in the last 5 years, using Boolean combinations of the terms “leadership,” “artificial intelligence,” and “human intelligence.” The thematic analysis allowed the identification of conceptual patterns and research gaps; the model elaborated from the review shows that leadership has an ethical and strategic mediation in the HI-AI relationship in a hybrid space of cooperation, in which automated decisions are put in real context through human judgment and reasoning; ethical governance mechanisms emerge for systems supported by artificial intelligence; and finally, a balancing mechanism to algorithmic efficiency is established through cognitive adaptability. The proposed framework offers organizations some guidelines for human supervision processes for AI-supported systems that integrate ethical evaluations into automated processes. It proposes elements—leadership tools that enhance the relationship between human intelligence and artificial intelligence. This article contributes to the management of organizations by proposing a model that recognizes leadership as a dynamic facilitator between HI and AI, integrating transdisciplinary knowledge of management, technological ethics, and cognitive science, and proposing an ethical interrelationship in the decision-making architectures between HI and AI. The proposed model establishes leadership mediation of human–AI interaction through four axes showing how leadership acts as the axis that brings together human and technological systems to work together. Hierarchical interaction creates a hybrid interaction that is highly flexible, efficient, and has ethical oversight. Finally, the proposed model is an open system that interacts with the environment and is understood as a flexible tool to support strategic decision-making in complex environments.

## 1. Introduction

Digital transformation processes have created a scenario where the boundaries between human and artificial capabilities are becoming increasingly blurred. This raises substantial questions about how organizations integrate artificial intelligence (AI) into organizational processes and how human intelligence (HI) relates to AI, particularly in complex organizational change, decision-making, innovation, and sustainability contexts. In this way, it is essential to know how leadership affects the interaction between human intelligence and artificial intelligence in situations where AI systems are being put in place quickly (Gignac & Szodorai, 2024; Russell & Norvig, 2010), without clear rules for governance and meaningful human involvement.

In this sense, leadership plays a role that can facilitate or hinder the articulation between both types of intelligence. Indeed, as has been described in recent years in the literature, how leaders conceive, adopt, and institutionalize intelligent technologies profoundly conditions collaboration, supervision, and delegation between humans and machines (Adeniyi et al., 2024; Floridi, 2019).

Since the 1950s, AI has evolved in its foundational concepts (McCarthy et al., 1955; Turing, 1950) to become a cross-cutting operational component during the last two decades in diverse sectors such as health (Hamet & Tremblay, 2017), finance (Bahrammirzaee, 2010), education (L. Chen et al., 2020), and security (Menouar et al., 2017). This expansion has sparked debates (Dellermann et al., 2019; Floridi, 2019; Sheikh et al., 2023) regarding its ethical limits, impact on employment, and the need for hybrid systems that integrate human capabilities in decision-making.

Since the beginning of the 20th century, human intelligence has been conceptualized from a psychometric perspective (Binet & Simon, 1904; Jensen, 1998) to use more comprehensive models that include emotional, social, and adaptive dimensions (Gardner, 2000; Salovey & Mayer, 1990; Sternberg, 1985). In recent years, studies have included in this conceptualization the importance of cognitive plasticity, experiential learning, and resilience as indicators of intelligent behavior in complex and changing environments (Kaplan & Haenlein, 2019; L. Meng et al., 2024; Oesch, 2024; Elfighi, 2025).

It can be said that the two forms of intelligence operate from different but complementary logics. At the same time, AI bases its functioning on algorithms, data processing, and statistical patterns (Chollet, 2017; LeCun et al., 2015); HI is built on interpretation, experience, and contextual judgment (Gignac, 2018; Plomin, 2019). However, the interaction between the two described intelligences is mediated by organizational structures, cultural norms, and leadership styles.

In a complementary way, leadership has been approached from different aspects, either as a relational, contingent, or multidimensional phenomenon (Bass & Stogdill, 1990; Northouse, 2010; Yukl, 2012). Likewise, some authors have described various types of leadership as effective in environments of accelerated technological change: transformational leadership (Tahir, 2015; Yahaya & Ebrahim, 2016), ethical leadership (Grover et al., 2022; Kumari et al., 2024), and adaptive leadership (Sharif & Ghodoosi, 2022). These types of leadership are relevant in environments where the role of the leader is not only to guide processes but also to act as a symbolic interface between the potential of AI and the human capabilities needed for its effective implementation (Dellermann et al., 2019).

Despite the studies described in the literature, there is evidence of a theoretical separation between studies on HI, AI, and leadership. Most works focus on pairs of these dimensions, such as AI and leadership or HI and leadership, without building models that integrate the three. The absence of an analytical framework that explains how leadership conditions the interaction between humans and AI represents a significant theoretical gap. To overcome existing gaps and contribute at a theoretical and practical level, this article aims to propose a conceptual model to help us understand how leadership affects human and AI interactions by combining different theoretical perspectives, finding points of convergence, and suggesting mediation scenarios that can help guide future research.

The proposed model establishes leadership mediation of human–AI interaction through four axes, showing how leadership acts as the axis that brings together human and technological systems to work together. Hierarchical interaction creates a hybrid interaction that is highly flexible, efficient, and has ethical oversight. Moreover, the model allows reconfiguring the functions of the area towards a more strategic and innovation-oriented approach to identify leadership profiles with a high capacity to manage human–machine teams, intervene in the design and implementation of training and development processes with an emphasis on digital, adaptive, and ethical skills, as well as the formulation of performance indicators that integrate human and technological criteria.

Finally, this model has a dual contribution. On the one hand, at a practical level, it is an open system that interacts with the environment and is understood as a flexible tool to support strategic decision-making in complex environments. On the other hand, at a theoretical level, it promotes the creation of hybrid models where human and artificial capabilities are articulated under human supervision or facilitation, which can impact organizational theories, sociotechnical systems, and organizational behavior. In other words, it raises the need to integrate knowledge aimed at developing new interdisciplinary theoretical frameworks that allow us to reformulate or expand existing theories to incorporate the idea that leadership not only influences people but also mediates the relationship between humans and autonomous systems.

## 2. Literature Review

### 2.1. Human Intelligence

Different theoretical frameworks, ranging from psychometric, dynamic, and contextual models, have traditionally explored the concept of human intelligence (HI). At the beginning of the 20th century, the first measurements of human intelligence were proposed through IQ tests, focused on verbal, spatial, and numerical skills (Binet & Simon, 1904; Jensen, 1998). However, the concept of multiple intelligences (Gardner, 2000) has expanded this vision. This idea includes different types of intelligence, like interpersonal, musical, kinesthetic, naturalistic, and intrapersonal. It also includes other approaches, like those that describe a combined view of analytical, creative, and practical aspects necessary for adapting to new situations (Sternberg, 1985).

On the other hand, Salovey and Mayer (1990) incorporate the concept of emotional intelligence, which is defined as the ability to perceive, understand, and regulate one’s and others’ emotions. EI has proven to be a significant predictor of effectiveness in leadership, conflict resolution, and decision-making (Goleman, 1995; Joseph & Newman, 2010).

From a neurological point of view, HI happens when parts of the brain, like the prefrontal cortex, hippocampus, and amygdala, interact in complicated ways. These parts help with memory, planning, and controlling emotions (Barbey et al., 2013). Current AI systems, which lack situational awareness and contextual judgment, are unable to fully replicate these processes.

Recent research has emphasized the relevance of cognitive plasticity as an emerging property of HI (Oesch, 2024; Pan et al., 2024); this plasticity is defined as the individual’s ability to adapt to new conditions through continuous learning. Competencies such as innovation, collaboration, and adaptive decision-making in organizational settings reflect their interpretation (Elfighi, 2025; L. Meng et al., 2024). Other studies describe the relationship between HI and ethical values, moral autonomy, and a sense of agency (Narvaez, 2002; Rest et al., 1999), elements that underpin legitimate and effective decision-making. Recent research has described HI’s ethical and relational characteristics as a necessary counterbalance to AI’s algorithmic efficiency (Glikson & Woolley, 2020). In conclusion, HI consists of integrated cognitive, emotional, ethical, and adaptive capabilities that enable individuals to make appropriate judgments of the environment, anticipate consequences, and act in context. These competencies are particularly valuable when AI cannot operate with moral sensitivity, outcome-based reasoning, or empathy.

### 2.2. Artificial Intelligence

The literature review conducted reveals a significant shift in the way AI has been conceptualized. Initial definitions were influenced by analogies with HI, as evidenced by the pioneering definitions of McCarthy et al. (1955) and Minsky (1968), who defined AI as a way of replicating human reasoning through logical machines. These definitions were articulated with computational models such as the Turing test (1950), in which they proposed conceptually and operationally evaluating the “intelligence” of a system based on its ability to simulate human conversation.

During the 1960s and 1970s, a symbolic-philosophical approach gained prominence, with authors such as Minsky (1968) delving into the idea that human thought could be represented through symbolic structures and logical rules. However, this paradigm encountered practical limitations when faced with problems of representing real-world knowledge. In parallel, the foundations of machine learning were established with models such as the perceptron, and the potential of simple neural networks was explored.

At the end of the 20th century and the beginning of the 21st century, the definition of AI shifted toward a vision more focused on adaptive behaviors. This perspective was described by Nilsson (1998), who proposed understanding AI as systems capable of behaving intelligently in complex environments. In this shift toward more functional models, Allen (1998) proposed that AI is centered on integrating perception, reasoning, and action, focusing on functional systems capable of operating in real-life environments. Therefore, as computational capabilities increased, new opportunities for deep learning opened.

Later, Russell and Norvig (2002, 2010) defined AI as the ability to act rationally and adapt to changing environments, going beyond the mere simulation of human thought. This was later incorporated with complex cognitive elements. For example, Legg and Hutter (2007) proposed a notion of AI based on the general ability to achieve goals through learning and adaptation, formulating a theoretical framework for AI. Also, LeCun et al. (2015) define AI as the ability of systems to learn hierarchical representations from large volumes of data, without the need for explicit programming.

Recently, Kaplan and Haenlein (2019) defined AI from an operational and practical perspective as the ability to interpret data, learn from it, and adapt to accomplish specific tasks. Chollet (2019) defines it as a system’s ability to acquire new skills and generalize efficiently from limited information, shifting the focus from observable behavior to the capacity for generalization. Wang (2019) emphasized that AI is the ability to adapt under resource constraints as an essential criterion.

For their part, Jiang et al. (2022) complement the previous definitions by highlighting that contemporary AI must be understood beyond technology, as a social and ethical phenomenon that requires comprehensive and interdisciplinary frameworks for its study and regulation. Likewise, Gignac and Szodorai (2024) highlight the operational nature of AI as an artificial emulation of human learning, reasoning, and decision-making processes, supported by algorithms and adaptive systems.

This evolution shows a transition from anthropomorphic notions to more pragmatic and technical understandings, evidencing the consolidation of AI as an autonomous field with its own theoretical identity.

The potential advances in AI development imply transparency, ethics, energy consumption, algorithmic justice, and security challenges (Floridi, 2019; van Wynsberghe, 2021). The European Commission (EC HLEG AI-European Commission, 2019) also thinks it is essential to set up governance frameworks in areas like health, education, defense, or public administration that focus on trustworthy, explicit use that is in line with social values (Holzinger et al., 2019).

HI and AI are different in ways that go beyond “logical reasoning” and “problem-solving.” These differences are in areas like “emotional intelligence,” “social cognition,” and “experiential learning” (Gardner, 2000; Sternberg, 1985). So, we should consider the connection between HI and AI as a dynamic complementarity that needs many mediators. Leadership is one of the most important factors for ensuring that both fields work well together.

### 2.3. Leadership

Leadership is one of the most explored topics in social and organizational sciences, due to its capacity to influence strategic processes, interpersonal relationships, and institutional outcomes (Northouse, 2010; Yukl, 2012).

Historically, the concept has developed from two fundamental visions: one focused on hierarchical leadership within organizational structures and another that perceives it as a process of social influence inherent in social systems (Yetton & Crouch, 1983). These visions have shaped four leadership theories: trait theory, behavioral theory, contingency theory, and transformational theory (Bruch & Walter, 2007). These theories have explored the characteristics and behavior of leaders, their exercise of power and influence, their adaptability in different contexts, and their management in changing business environments (Prabhu & Srivastava, 2023). None of these approaches is mutually exclusive or limited to a specific period (Al-Taneiji, 2006).

The theoretical development of leadership has been marked by a shift from approaches focused on individual characteristics to approaches that recognize the importance of behavior, context, and contingency. Some theories (Goleman, 2004; Griffin & Moorhead, 2013; Henkel & Bourdeau, 2018) focused on identifying traits inherent in ineffective leaders, such as intelligence, self-confidence, resilience, and strategic vision. These theories demonstrated their limitations by explaining that possessing personal attributes (Yukl, 2006). This process led to the formulation of behavioral theories centered on observable leader behavior patterns.

Other research, such as that developed at Ohio State University and the University of Michigan, identified two fundamental dimensions of leadership behavior: task orientation and people orientation (Blake & Mouton, 1985; Katz et al., 1951; Stogdill & Coons, 1957). The description of these approaches led to the conclusion that leadership effectiveness may depend on a balance between achieving goals and the quality of interpersonal relationships (Baczyńska & Thornton, 2017; Fambrough & Hart, 2008). Other authors (Vecchiotti, 2018) have argued that the relationship between leaders and followers is a central element for organizational success and that the cultural and institutional context mediates this relationship.

From a purely conceptual perspective, current definitions of leadership converge on three key elements: influence, group management, and a focus on shared goals. Leadership is a relational process within a social group, through which a person influences others to achieve common goals (Northouse, 2010; Yukl, 2006, 2012). This influence can manifest through the explicit exercise of authority or arise through the social attribution of legitimacy based on the perception of followers (Ladkin, 2010; Lord & Maher, 2002).

Thus, leadership is not a static property of the individual but situated practices shaped by social interaction and the organizational context (Blake & Mouton, 1985; Rue & Byars, 2005). This is consistent with the argument that a key function of leadership is to focus the collective’s attention on strategic problems, articulating visions that give meaning and direction to joint action (Cyert, 1990). Other aspects, such as the knowledge of the leader’s value system, vision, influence, and followers’ presence, are recurrent in multiple definitions, suggesting a shared conceptual structure and being considered determinants in the configuration of leadership style and behavior (Han, 2015; Sosik, 2005; Vroom & Jago, 2007).

Currently, among the most influential leadership typologies are “transformational leadership,” “ethical leadership,” and “adaptive leadership.” The first type of leadership involves inspiring, motivating, and developing team members to perform at higher levels (Bass, 1985; Tahir, 2015). This leadership style has been shown to work well in situations that require innovation and organizational change, mainly when disruptive technologies like AI are used (Dellermann et al., 2019).

On the other hand, ethical leadership focuses on integrity, equity, and orientation towards the common good in decision-making. It is relevant in managing technologies with ethical implications (Brown & Treviño, 2006; Grover et al., 2022), such as using algorithms in high-impact decisions or supervising automated systems that can replicate biases (Floridi, 2019; Kumari et al., 2024).

In addition, adaptive leadership examines how leaders can deal with non-technical problems, create meaning for everyone, and help their teams learn in uncertain situations (Heifetz et al., 2009; Sharif & Ghodoosi, 2022). This method works well when HI and AI are not fully integrated with standard protocols.

In summary, one of the key functions of contemporary leadership is to serve as a mediator between technical and human dimensions, particularly in automation processes. The leader acts as a “sense-maker,” facilitating collective understanding of change, managing emerging emotions, and constructing narratives that legitimize the responsible use of AI (Goleman, 1995; Weick, 1995; Jarrahi, 2018).

The literature recognizes leadership as critical in configuring collaborative scenarios between humans and machines. Empirical studies (Glikson & Woolley, 2020) show that leadership style influences trust in intelligent systems, the willingness to use AI, and the quality of human–algorithm decisions. Leadership also modulates the relationship between HI and AI, from a logic of supervision to a logic of association or co-creation.

## 3. Materials and Methods

This research uses an integrative literature review research strategy, which is structured but not systematic. It is a methodical and thorough way of gathering information about research methods, theories, and results in a conclusion about a particular subject (Guirao, 2015). This enabled us to delineate the current state of knowledge concerning the interplay between “human intelligence” (HI), “artificial intelligence” (AI), and “leadership”.

Following Gómez-Luna et al. (2014), a research topic was chosen, high-quality databases were used, the data were organized by theme and time, and the most important studies and contributions were put together. Finally, we critically reviewed the documents to consolidate the results.

This thematic delimitation allowed for the construction of a line of knowledge consistent with the study’s objectives, avoiding deviations into collateral or marginal publications that could dilute or generate confusion (Ferrari, 2015).

The central inclusion criterion was a substantive contribution to the theoretical debate. Studies that offered explanatory models, definitions of classic works, and current developments on HI, AI, and leadership, as well as studies on transformational, ethical, and adaptive leadership, were considered especially valuable. This theoretical richness allowed for the development of conceptual frameworks for each topic and the identification of emerging proposals.

Inclusion criteria: Publications with an explicit focus on at least two of the three dimensions studied: HI, AI, and leadership.

The exclusion criteria were guided by sources without academic peer review, studies focused exclusively on AI technical development without human-organizational links, and publications of poor editorial quality or not unverifiable references.

The literature review was based primarily on peer-reviewed publications, academic book chapters, and technical studies from recognized scientific institutions, provided they had clear institutional traceability.

Standardized methodological evaluation tools such as AMSTAR or PRISMA were not applied, given the narrative nature of the review. However, the researchers exercised rigorous critical judgment, discarding sources with insufficient foundation, without references, or those that presented obvious biases or speculative conclusions without empirical or argumentative basis. Above all, the sources were sought to offer conceptual solidity, clarity of exposition, and recognition within the academic community (Byrne, 2016).

Interdisciplinary sources were included to capture the complexity and transdisciplinarity of the phenomenon studied (Ferrari, 2015). To reduce the number of sessions, the review includes theoretical and empirical studies and proposals for technological applications in educational, corporate, clinical, and governmental contexts. The variety of perspectives and methodologies allowed the research team to contrast perspectives and enrich the understanding of the links between HI, AI, and leadership from multiple epistemic frameworks.

It recognizes the exclusion of publications in languages other than English and Spanish as a main limitation, which may limit the geographical and cultural diversity of the approaches. A bias toward theoretical currents more represented in the West is also possible, given the predominance of North American and European sources in the databases consulted. Furthermore, the narrative selection implies subjectivity in the evaluation, categorization, and weighting of sources, although this was compensated for through an iterative and triangular analysis process among authors.

The search for bibliographic sources was conducted in the scientific databases Scopus and Web of Science, selected for their multidisciplinary coverage and high level of rigor. The publication period considered was the last 5 years to capture recent transformations in the field, especially those derived from the rise in generative AI and the post-COVID-19 pandemic impact on organizational dynamics. The research process starts with the hypothesis of theoretical gaps and emerging convergences based on an analytical review of the academic literature from 2020 to 2024, including the first quarter of 2025, and the relevant texts. For the search, the terms human intelligence (HI) and artificial intelligence (AI) were integrated into human–AI.

The following terms and their Boolean combinations were used as search axes: “leadership,” “human-AI,” “human-AI interactions,” “human-AI collaboration,” AND “human-AI,” and “leadership”, AND “human-AI interactions,” AND “human-AI collaboration.” This structure allowed us to identify studies that addressed each dimension separately and research that explored their cross-relationships.

Articles that were (a) reviewed by experts in the field; (b) listed in Q1, Q2 journals, and other high-impact journals, and (c) research that focused on the interaction between people and machines in business settings were all considered.

Duplicate sources, purely technical documents with no organizational implications, and publications lacking explicit theoretical frameworks were excluded. As a result, 408 articles were found, of which 323 were selected.

The literature review helped us come up with three main conclusions. First, most studies examining the relationship between human intelligence and AI only look at cognitive complementarities, not governance frameworks or the symbolic aspect of leadership (Dellermann et al., 2019; Glikson & Woolley, 2020). Second, there is abundant literature on leadership and technological innovation, although few studies analyze the role of leadership as an explicit mediator between AI and HI (Setyaningrum & Muafi, 2023). Third, works that address the three variables in an integrated way tend to be exploratory, with little suggestion of formalized analytical models.

The models proposed in this study provide tools to close the gaps described in the literature in an integrated and coherent manner, particularly those related to the articulation of hybrid roles, processes, and specific leadership behaviors in human–AI teams. The model proposed in this study offers a structure that facilitates the visualization of the leader’s roles beyond the instrumental or technical, serving as a central node of the organizational processes in which artificial intelligence plays an active role, facilitating its integration into organizational management. In this sense, competencies such as the interpretation of algorithmic data (Chollet, 2019), the empathic communication of automated decisions (L. Meng et al., 2024), ethical understanding, and the facilitation of adaptive processes (Ebojoh & Högberg, 2024) emerge as innovative elements in addition to traditional leadership models.

**Figure 1:**
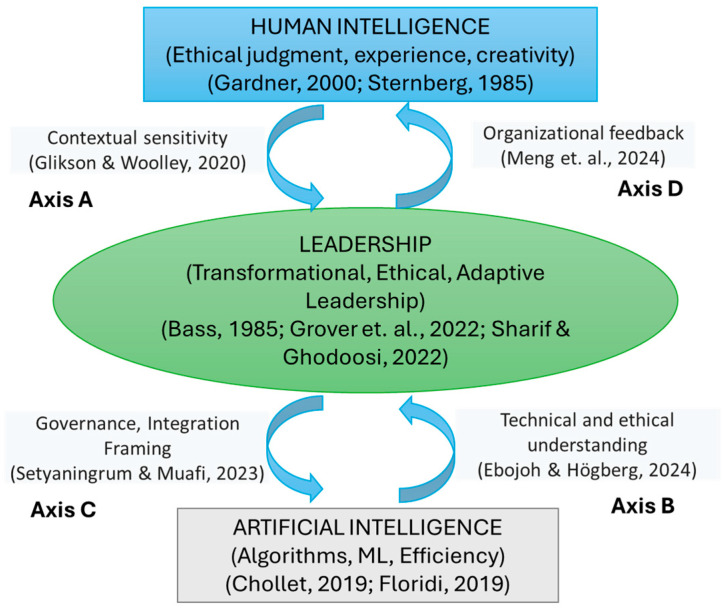
Leadership mediation model 1 in human–AI interaction.

In addition, the literature review revealed a gap in knowledge regarding the bidirectional relationship between human intelligence and artificial intelligence. The literature describes the relationship between human intelligence and artificial intelligence, focusing on how AI adapts to human patterns (Minsky, 1968; Ford, 2018) or on the mechanisms of human oversight of algorithmic decisions (Cath et al., 2017; Hermansyah et al., 2023). However, the proposed model 1 introduces a bidirectional feedback relationship that analyzes people’s adaptability to AI, as well as changes in human practices resulting from co-learning. Leadership is the mediating variable, as shown in the B/D axes (Figure 1). This approach raises the need to develop cognitive, emotional, and ethical competencies in both leaders and their teams to effectively address this dual transformation.

Along these lines, the proposed model 1 (Figure 1) includes interaction paths in which leadership becomes a catalyst for organizational learning (L. Meng et al., 2024), which facilitates the adoption of intelligent systems focused on the ethical and functional understanding of technology (Ebojoh & Högberg, 2024) and establishes a guide for adaptive interaction with human behavior, generating organizational feedback that promotes the continuous improvement of intelligent systems based on human experience, as shown in the A/C axes (Figure 1).

**Figure 2:**
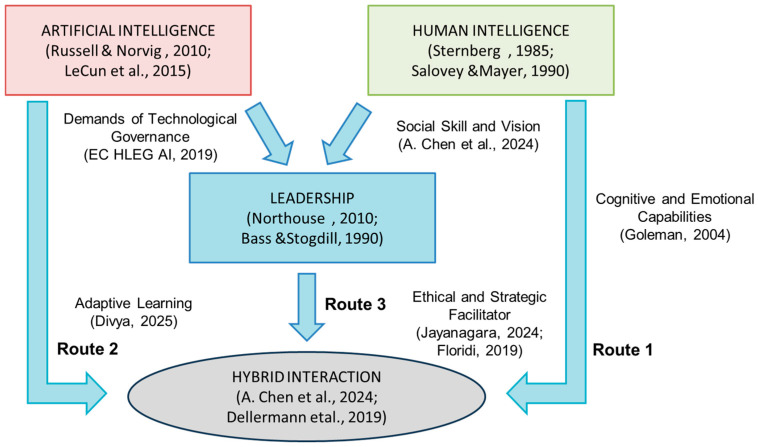
Leadership mediation model 2 in human–AI interaction.

The model 2 (Figure 2) proposes elements that give leadership a role as a strategic mediator for conflict management (Adeniyi et al., 2024), particularly those derived from the perception of AI as a competitor to human functions and from the flexible hierarchical interaction in which the roles associated with each of the human–artificial intelligences are divided (Peltonen & Topaz, 2022). In the long term, it is expected that, as proposed by Parry et al. (2016), this favors the development of resilient organizational cultures, with less resistance to change and greater clarity in the functional limits of each actor (human or artificial).

Due to this conceptual gap, it is necessary to propose a conceptual model that explains the ideas from a theoretical perspective and can be utilized to inform strategic decisions for public, private, and third-sector organizations.

In this way, the model we will talk about in the results section is based on the parts we found in the review and reorders them based on three factors: (1) how the human–AI interaction works, (2) the most common leadership styles, and (3) the specifics of the organization.

It should be noted that although this review does not adopt the methodology of a systematic literature review, it does apply principles of traceability, transparency, and thematic analysis that allow us to guarantee the argumentative coherence of the proposed model. In addition, an iterative approach was used in the reading and categorizing of sources, allowing a progressive coding of patterns, key concepts, and relationships between variables.

## 4. Results

This study describes an integrative literature review and suggests the “Leadership Mediation Model in Human-AI Interaction,” in which leadership acts as a dynamic mediator in the relationship between human intelligence and artificial intelligence.

### 4.1. Leadership Mediation Model in Human–AI Interaction

This model integrates three structural nodes: Node1, “Human Intelligence” (HI), which refers to judgment, experience, ethics, and adaptability; Node2, “Artificial Intelligence” (AI), which contains algorithms, machine learning, and efficiency; and a third node, “Leadership,” which mediates between the two previous ones (Figure 1).

The model reflects the following axes of influence: Axis A: From HI to leadership requires contextual sensitivity (Glikson & Woolley, 2020; Vargas, 2025); Axis B: From AI to leadership—demands technical understanding and ethical vision (Tiwari et al., 2022; Parchment, 2025; Gupta & Jaiswal, 2024; Baez, 2025; Bevilacqua et al., 2025); Axis C: From leadership to AI—demands governance and an integration framework (Alshaibani et al., 2025); Axis D: From leadership to AI regarding organizational feedback (Rukadikar & Khandelwal, 2024; Hossain et al., 2025; Florea & Croitoru, 2025; Bock & von der Oelsnitz, 2025).

#### Conceptual Model of Hybrid Interaction

Along with the first model (Figure 1), the documentary analysis helped solidify a second hybrid interaction model that includes the interaction between leadership and human–AI. This model shows how leadership acts as the axis that brings together human and technological systems to work together. Hierarchical interaction creates a hybrid interaction that is highly flexible, efficient, and has ethical oversight.

The second model (Figure 2) highlights three main routes of interaction:

Route 1 from HI towards hybrid interaction, focusing on the cognitive, social, and emotional capabilities that enrich decision-making processes (Goleman, 2004).

Route 2 involves a shift from AI to hybrid interaction, which relies on computational capabilities to enable large-scale information processing and predictive analysis (LeCun et al., 2015; Russell & Norvig, 2010).

Route 3 from leadership toward hybrid interaction, assuming a role of strategic, ethical, and transformational mediation (Russell & Norvig, 2010; Jayanagara, 2024; Northouse, 2010). As shown in Figure 1, this model also considers how leadership can affect both HI (e.g., informative and adaptive settings) and AI (e.g., in terms of governance and ethical orientation). Recent research has emphasized these connections (A. Chen et al., 2024; EC HLEG AI-European Commission, 2019).

### 4.2. Applicability of the Proposed Model

The proposed model can be used as an analysis framework to design people-centered AI implementation policies, train leaders with hybrid skills (technological and ethical), and diagnose organizations’ maturity levels in the face of automation processes. In the same way, the model lets you find the most important parts of combining human–AI and suggests actions that leaders can take to maximize their strengths and reduce the risks to the whole system.

In the strategic management of human talent, the model allows reconfiguring the functions of the area towards a more strategic and innovation-oriented approach to identify leadership profiles with a high capacity to manage human–machine teams, intervene in the design and implementation of training and development processes with an emphasis on digital, adaptive, and ethical skills, as well as the formulation of performance indicators that integrate human and technological criteria.

In automation processes, the model can serve as a guide to evaluate the relevance, scope, and limits of the deployment of intelligent technologies. Under this approach, leadership guarantees the balance between operational efficiency and human sustainability, establishing supervision mechanisms that maintain human agency as the central axis of digital transformation.

Finally, the model allows for identifying and evaluating cultural barriers, symbolic resistances, and ethical gaps that could affect the legitimacy of change. As a facilitator, leadership can reduce these tensions and turn them into opportunities for dialog, organizational learning, and shared meaning construction.

It is relevant to clarify that the proposed model is an open system that interacts with the environment and is understood as a flexible tool to support strategic decision-making in complex environments.

One of the model’s most relevant contributions is the incorporation of explicit mechanisms for leadership to ensure algorithmic transparency through ethical communication frameworks (Zarsky, 2016), to close the gap in the approach taken in the literature regarding the communication of decisions made based on AI algorithms and the trust and transparency derived from them (Akinrinola et al., 2024; Wu et al., 2024; Wu et al., 2025; Q. Meng et al., 2025). This involves translating automated decisions into languages understandable to teams, establishing criteria to measure trust in AI, and defining adequate levels of explainability without compromising the efficiency of the system (Balasubramaniam et al., 2022). These practices can strengthen the legitimacy of AI in the organizational environment (Thiebes et al., 2021).

## 5. Discussion

### 5.1. Interaction Between Human–AI

Multiple factors have driven recent advances in machine learning. For starters, there have been significant theoretical advances in machine learning methods like decision trees (Quinlan, 1986), support vector machines (Cortes & Vapnik, 1995), random forests (Breiman, 2001), AdaBoost (Freund & Schapire, 1997), and better neural networks through error backpropagation (Rumelhart et al., 1986). Subsequently, introducing the ’dropout’ technique (Hinton et al., 2012) helped mitigate overfitting in neural networks.

Second, global interconnection and the availability of vast data have made it possible to train complicated algorithms (Givigi & Jardine, 2018; Jiang et al., 2022; Li & Bitterly, 2024; Zhang et al., 2018). Thirdly, advancements in accelerated computing through GPUs, NPUs, and specialized architectures have enabled computations to be completed in less time, which previously might have taken months or years (Kelly, 2016; Meidan et al., 2011; Sun et al., 2019; Yin et al., 2015; Zaman et al., 2022).

These factors have significantly contributed to developing more sophisticated and efficient models in machine learning, enabling innovative applications in various areas. These advances have made it possible to create more robust and versatile systems capable of processing large volumes of data and performing complex tasks with greater accuracy and speed.

The human–AI interaction becomes fundamental to providing an ethical framework for the use of results and establishing supervisory frameworks for automated decision-making systems; that is, in functional terms, human intelligence contributes elements such as contextual judgment, ethical sensitivity, divergent creativity, and empathy, while artificial intelligence offers speed, statistical accuracy, massive processing capacity, and algorithmic consistency (Chollet, 2019; Floridi, 2019).

This relationship is not dichotomous but complementary; its effectiveness depends on the implementation context, organizational objectives, and the leadership style that structures this interaction (Alshaibani et al., 2025; Baez, 2025; Bevilacqua et al., 2025; Bock & von der Oelsnitz, 2025; Florea & Croitoru, 2025; Vargas, 2025; Hossain et al., 2025; Gupta & Jaiswal, 2024; Rukadikar & Khandelwal, 2024; Tiwari et al., 2022).

In addition, the literature talks about how augmented intelligence (IAu) has grown along with artificial intelligence (AI), becoming abilities used with computers (Hassani et al., 2020). This approach has been around since 1962 (Engelbart, 1962; Skagestad, 1993; Skagestad, 1996) when it first came up. It has been considered a middle ground between full automation and only human skills (Kyllonen et al., 2008), putting people at the center of technology interactions (101). Although the discursive dominance of AI has overshadowed its relevance (Hassani et al., 2020), both concepts converge in applications that combine algorithmic analysis with human judgment. Thus, the idea of hybrid models appears in human–machine cooperation.

This idea states that hybrid intelligence systems are like collaborative architecture that balances the freedom of algorithms with oversight from humans (Dellermann et al., 2019; Pileggi, 2024). These models combine traditional AI methods and new strategies to make them easier to understand and avoid problems that have happened in the past when making decisions (Rizvi, 2023; Yu, 2024).

AI can be used in strategic areas like defense (Preece et al., 2019), where cognitive synergies can improve operational processes, and in natural resource management, where predictive criteria can be used together (Zaresefat & Derakhshani, 2023) to make better decisions. While combining deep learning with symbolic intelligence has significantly improved medical diagnosis (Musanga et al., 2024), combining data-driven models with natural language systems makes technology more flexible (Maletzki et al., 2024).

However, despite the opportunity these authors propose, others consider that the ethical challenges and the absence of robust regulatory frameworks that support implementing these technologies must be highlighted (Raisch & Krakowski, 2021). To this end, effective governance must balance operational efficiency with moral integrity, particularly in sensitive environments such as financial audits (Homayounirad, 2023) or educational systems (Bredeweg & Kragten, 2022). Other research suggests using transparency architectures that connect automated processing with accountability protocols (Schuering & Schmid, 2024) to ensure that hybrid decisions can be tracked (Li & Bitterly, 2024; van der Waa et al., 2022).

### 5.2. Influence of Leadership on Human–AI Collaboration

Leadership is a mediating variable that conditions the relationship between human–AI. Transformational leadership creates spaces where people are open to learning and trusting technology; ethical leadership ensures that AI is used in a fair, honest, and responsible way. In addition, adaptive leadership lets people and algorithms change how they work together based on new problems (Grover et al., 2022; Sharif & Ghodoosi, 2022).

This mediating effect shows up on three levels: (1) in the way human and intelligent systems work together; (2) in how ethical and communicational frameworks make the use of AI seem reasonable; and (3) in how well organizations can adapt to automation processes without losing their ability to use good judgment.

The success of hybrid intelligence ecosystems depends on leadership models that explain how people and technology interact (Dellermann et al., 2019). This implies dual functions: interpreting algorithmic results in organizational contexts and establishing ethical protocols to mitigate biases (Dave & Mandvikar, 2023). Experience in manufacturing and services demonstrates that strategic mediation increases productivity and job satisfaction by redistributing repetitive tasks toward creative roles (Preece et al., 2019).

In the future, the convergence between augmented and hybrid AI tends to define innovation paradigms, requiring a transdisciplinary approach that integrates computational sciences, applied ethics, and organizational theory; in this sense, some researchers (van Breemen et al., 2011; Yu, 2024) highlight the need to develop standardized metrics to evaluate the socio-technical impact of these systems, particularly in their capacity to enhance and not replace human agency.

## 6. Conclusions

This study proposes a conceptual model that explains the influence of leadership on the human–AI relationship. According to the literature review over the last 5 years, the key to a long-lasting and moral combination of human–AI is good algorithm design, advanced technology, and the kind of leadership that guides this relationship. Transformational, ethical, and adaptive leadership committed to innovation and human well-being is required to facilitate this relationship.

The literature particularly highlights the role played by transformational leadership as a style characterized by its ability to inspire, mobilize, and generate a shared vision. It establishes conditions of trust that are indispensable in automation, digitalization, and adoption of disruptive technologies (Bass & Riggio, 2006; Cyert, 1990), making it the most conducive leadership style to articulate collaborative relationships between HI and AI.

The information in this article led to the creation of a new conceptual model that helps us understand the role of leadership in human–AI interaction. The model was based on an updated literature review and an in-depth analysis of specialized literature. It shows that leadership helps humans and machines become more technical and cognitively similar, and it is an important part of establishing this relationship because it serves a normative, ethical, and symbolic purpose.

The proposed models show that good leadership can help technological integration strategies happen. These initiatives can lead to changes in production processes and more resilient and adaptable organizational cultures (Glikson & Woolley, 2020; Setyaningrum & Muafi, 2023).

This model can be used in business settings in three ways: (1) Change management, where leaders use inclusive and caring stories to reduce resistance to AI. (2) Organizational innovation, by creating conditions that make it easier for humans and algorithms to work together; and (3) Technological governance, by setting up ethical frameworks and procedures that guide the proper use of AI (Floridi, 2019).

This proposal corresponds to the five leadership practices model (Kouzes & Posner, 2017). These five practices enhance leadership effectiveness in the human–AI interaction, providing them with the necessary tools to manage situations with complex technology and adaptive uncertainty. Therefore, the proposed model is grounded in contemporary organizational theories and applied and replicable leadership frameworks.

In summary, this work provides an integrative theoretical foundation linking three traditionally separate domains—human intelligence, artificial intelligence, and leadership—offering a useful tool for reflection, institutional design, and strategic action. Future lines of research should focus on empirically validating this model, adapting it to specific sectors as described in the literature, and exploring its applicability in multicultural contexts.

### 6.1. Limitations

The choice of a narrative review, rather than a systematic review, arises from the need to capture the theoretical complexity, historical evolution, and interdisciplinary tensions that exist in the articulation between HI, AI, and leadership. However, it is recognized that this methodological decision entails epistemological limitations.

One of the main limitations is the potential biased selection of sources, as this method is mediated by the experience, judgment, and focus of the research group (Ferrari, 2015). Narrative reviews are based on an interpretive judgment that, while informed, can lead to inadvertent omissions or an overemphasis on theoretical approaches or the conceptual and operational definitions of analytical variables.

Furthermore, the narrative review presents a potential bias toward normative perspectives of leadership, such as transformational leadership (Bass, 1985; Yukl, 2008) or ethical leadership (Brown & Treviño, 2006), to the detriment of critical or counter-hegemonic perspectives. This tendency may limit the explanatory scope of the study when addressing organizational realities in which AI implementation does not respond to collaborative approaches, but rather to dynamics of power, exclusion, or surveillance, characteristic of other leadership theories, such as transactional leadership.

On the other hand, the geographical origin of the sources may introduce bias into the review. Although the inclusion criterion was language (English and Spanish), most of the publications are North American and European, reflecting a cultural and contextual bias. Studies from other geographical areas, such as Asia, Africa, or Latin America, on leadership in AI environments are scarce in the review, either due to language barriers or their lower visibility in indexed databases.

These limitations and biases, although inherent to the narrative review, do not invalidate the study’s findings. On the contrary, they reinforce the need to adopt a critical and reflective stance regarding one’s methodological decisions. They also seek to promote the development of future research with geographical approaches and complex, transdisciplinary theoretical developments.

In short, the novelty and evolution of these concepts and their theoretical frameworks may affect the generalizability of the conclusions. While these concepts and the relationships proposed in this study offer an innovative framework, their development is still incipient, requires empirical validation, and is not free from theoretical controversies that will need to be addressed in future research.

### 6.2. Future Lines of Research

Human–AI interaction models recognize that leadership becomes a symbolic and operational element for mediating between people and intelligent systems at the ethical, strategic, and adaptive levels. In this context, the leader becomes a mediator between HI and AI, as well as a guarantor of regulatory frameworks to regulate the implementation of algorithmic technologies without displacing human agency. This approach opens a future line of research aimed at delineating the specific roles of leadership in environments of interaction between HI and AI, as well as the discursive, symbolic, and structural communication skills that enable leaders to generate hybrid collaborative environments.

Since leadership, according to the proposed model, is a mediating element, it is necessary to expand research to reestablish leadership styles that foster greater trust and a sense of agency in teams when interacting with AI. Inspired by Morin’s (2001) complexity thinking and Luhmann’s (1998) systems theory, the models presented articulate various levels of analysis to interpret the interaction between HI, AI, and leadership. Morin (2001) proposes overcoming fragmented thinking through a logic that assumes uncertainty, interdependence, and feedback as epistemological principles. Specifically, it paves the way for research that develops explanatory models that consider dynamic, nonlinear, adaptive, and evolutionary interactions between human actors, intelligent artifacts, and institutional environments.

In parallel, Luhmann (1998) proposes that social systems operate through functional differentiation. In these systems, in line with the proposed model, leadership can act as a node that fosters structural coupling between the technical system (AI) and the human system (organization and culture). This approach promotes a line of research focused on the construction of transdisciplinary theoretical frameworks (Nicolescu, 2006), which integrate the epistemology of leadership, second-order cybernetics, augmented intelligence, and general systems theory.

## Data Availability

The databases used in this study are available at Scopus https://www.scopus.com/, and Web of Science https://www.webofscience.com/ (accessed on 12 March 2025).

## Abbreviations

The following abbreviations are used in this manuscript:

HI	Human Intelligence
AI	Artificial Intelligence
